# Balancing the Interests of Patient Data Protection and Medication Safety Monitoring in a Public-Private Partnership

**DOI:** 10.2196/medinform.3937

**Published:** 2015-04-15

**Authors:** Nancy A Dreyer, Stella Blackburn, Valerie Hliva, Shahrul Mt-Isa, Jonathan Richardson, Anna Jamry-Dziurla, Alison Bourke, Rebecca Johnson

**Affiliations:** ^1^Quintiles Real-World & Late-Phase ResearchScientific AffairsCambridge, MAUnited States; ^2^Quintiles Real-World & Late-Phase ResearchReadingUnited Kingdom; ^3^Quintiles Real-World & Late-Phase ResearchSt. PrexSwitzerland; ^4^School of Public HealthImperial College LondonLondonUnited Kingdom; ^5^Newcastle UniversityNewcastle upon TyneUnited Kingdom; ^6^Poznan University of Medical SciencesPoznanPoland; ^7^Cegedim Strategic DataLondonUnited Kingdom; ^8^International Alliance of Patients’ OrganizationsLondonUnited Kingdom

**Keywords:** pharmacovgilance, pregnancy, Internet, public-private partnerships, data protection, ethics

## Abstract

Obtaining data without the intervention of a health care provider represents an opportunity to expand understanding of the safety of medications used in difficult-to-study situations, like the first trimester of pregnancy when women may not present for medical care. While it is widely agreed that personal data, and in particular medical data, needs to be protected from unauthorized use, data protection requirements for population-based studies vary substantially by country. For public-private partnerships, the complexities are enhanced. The objective of this viewpoint paper is to illustrate the challenges related to data protection based on our experiences when performing relatively straightforward direct-to-patient noninterventional research via the Internet or telephone in four European countries. Pregnant women were invited to participate via the Internet or using an automated telephone response system in Denmark, the Netherlands, Poland, and the United Kingdom. Information was sought on medications, other factors that may cause birth defects, and pregnancy outcome. Issues relating to legal controllership of data were most problematic; assuring compliance with data protection requirements took about two years. There were also inconsistencies in the willingness to accept nonwritten informed consent. Nonetheless, enrollment and data collection have been completed, and analysis is in progress. Using direct reporting from consumers to study the safety of medicinal products allows researchers to address a myriad of research questions relating to everyday clinical practice, including treatment heterogeneity in population subgroups not traditionally included in clinical trials, like pregnant women, children, and the elderly. Nonetheless, there are a variety of administrative barriers relating to data protection and informed consent, particularly within the structure of a public-private partnership.

## Introduction

### First Do No Harm

The Declaration of Helsinki extends the ancient medical tenet of “*Primum non nocere”*, “first do no harm”, and provides protection to human subjects of medical research by establishing ethical principles to ensure that medical research can never take precedence over the rights and interests of individual research subjects [1]. While laudable, harm can also occur by over-zealous interpretation of rules and regulations that overcomplicate studies, while adding little, or nothing, to the protection of subjects. The European Union (EU) “EU Data Protection Directive” by the European Commission (EC) (European Directive 95/46 EC) was intended to enable personal data “to flow freely from one Member State to another”, while safeguarding the fundamental rights of individuals, yet its implementation into national law has given rise to a myriad of interpretations, making multi-country studies challenging.

### Medication Safety in Pregnancy

Consider, as an example, the importance of understanding which medications can be safely used during pregnancy, especially during the first trimester, since some exposures at this time may have teratogenic potential [2,3]. Inclusion of pregnant women in preclinical randomized controlled trials is generally considered unethical due to the unknown risks which may be posed to the developing fetus, and as such, pregnant patients are often excluded unless the medicine is specifically for a pregnancy related condition. Consequently, safety data for pregnancy outcomes must be collected after licensing via noninterventional observational studies, which often utilize pharmacoepidemiologic techniques to analyze large databases, such as electronic health records to look for rare events such as specific congenital anomalies. However, these databases may not contain information about lifestyle and other factors, which may also affect the outcome of pregnancy, or may not contain adequate details concerning concomitant risk factors. These omissions could bias study interpretation. Hence, the development and testing of alternative methods of data collection for pharmacovigilance is important.

Here, we describe the legislative challenges in data ownership and barriers to approval faced by a public-private partnership in conducting an observational study of self-reported maternal medication use and pregnancy outcomes.

## Our Experiences

### Example of Challenges of Data Ownership and Barriers to Approval

This observational study of direct-to-consumer data collection on various exposures during pregnancy was conducted through a public-private partnership known as the Pharmacoepidemiological Research on Outcomes of Therapeutics by a European ConsorTium (PROTECT) [4], which was coordinated by the European Medicines Agency. The PROTECT project received support from the Innovative Medicines Initiative (IMI) Joint Undertaking, which included financial contribution from the EU's Seventh Framework Programme (FP7/2007-2013) and in-kind contribution from the European Federation of Pharmaceutical Industries and Associations. PROTECT consisted of 35 partners including pharmaceutical companies, academic organizations, national and international regulatory agencies, patient organizations, and other interested parties, and the IMI has now extended this public-private partnership model to address other important public health concerns [5].

While other PROTECT work packages focused on methodological challenges using existing data sources, we explored digital technologies for frequent and timely data collection from consumers for the purposes of determining whether this is a viable alternative as a pharmacovigilance tool.

This study was conducted according to the current best practices for noninterventional drug safety research including full protocol, specification of analytic methods and data to be collected, and a description of the plan for protecting human subjects [6]. Pregnant women were invited to participate via Internet or using an automated telephone response system (Interactive Voice Response System). Information was collected via a secure website from women in Denmark, the Netherlands, Poland, and the United Kingdom (UK) who identified themselves as pregnant, and were recruited through websites, emails, leaflets, television, and social media platforms. Health care professionals were not involved directly in study recruitment or promotion. Data were collected on prescription, nonprescription and herbal medications, recreational drug use, age, ethnicity, and lifestyle factors. Data were treated with strict confidential measures; for example, contact details were key-coded and deleted after the study end, and medical data were stored on a separate, secure server with restricted physical and password access. Local academic centers and a national health system entity served as country study leads, and notified the local ethics committee and data protection agencies. Regulatory and data protection submissions were performed according to the local requirements in the participating countries.

### Some Examples of Variations by Country

There was substantial variation in the requirements for ethical review. Table 1 shows the differences in protocol requirements and the length of time needed for ethical and data protection review in each country and by the European Medicines Agency.

**Table 1:** Country specific protocol differences; ethical and data protection requirements and timing.

Protocol differences	Denmark	Netherlands	Poland	United Kingdom	European Medicines Agency
Country lead	Statens Serum Institute	University of Groningen	Poznan University of Medical Sciences	Newcastle University	N/A^a^
Minimum age (years)	18	18	18	16	N/A^a^
Informed consent	Electronic only, IVRS^b^ not acceptable	Both Internet and IVRS^b^ possible	Written informed consent required in addition to Internet and IVRS^b^ informed consent	Both Internet and IVRS^b^ possible	N/A^a^
Consent for individual record linkage	Required for study entry	N/A^a^	N/A^a^	Separate consent requested	N/A^a^
Ethical approval timing	Not required	Waiver (certificate of nonobjection)	1 week	3 weeks	N/A^a^
Time for data protection approval	~3 months	1 day	9 months	2 weeks	3 months opinion, 5 months prior check

^a^ N/A=not applicable

^b^ IVRS = Interactive Voice Response system

### Some Examples of Variations by Country

Denmark did not require ethical review for an observational study. In the Netherlands, a waiver (literally, a certification of nonobjection) was granted since the personal identifiers were securely retained and maintained separately from study analysis files. In Poland and the UK, ethics submission required submitting the study protocol and all study documents (informed consent, questionnaires, etc) and other administrative information.

It is also worth noting the differences between countries in enrollment requirements and informed consent. Although the study was designed to give the choice of participating by phone or Internet to facilitate recruitment of low-income women, one country required all participants to enroll on the Internet before being able to respond by phone, and another required printing and mailing written informed consent in addition to consent by phone or Internet.

Formal notifications were required for data protection. The European Medicines Agency, as required under Article 27 of Regulation (EC) number 45/2001, submitted a notification for prior check with the European Data Protection Supervisor (EDPS) in October 2010. The EDPS opinion was that, since all study partners were involved in the development of the protocol and all could decide on the “means and purposes of the processing of personal data” and review results, all study partners effectively determined the purposes of the collection of the data and were “joint controllers”. As a result of this ruling, a formal memorandum was prepared detailing each partner’s role and participation in the study, responsibilities to the study and other partners, and to data protection. It took about 14 months to get these agreements in place, since they required agreement from all study partners. After these provisions were in place, the EDPS confirmed that the processing operations would not involve any breach of Regulation (EC) No 45/2001.

In the Netherlands, approval of data protection was granted on the same day the request was submitted, and review was also relatively quick in the UK and in Denmark. However, review by the Polish Data Protection Agency took 9 months and required submission of special items including the characteristics of the Personal Data Administrator, the technical and organizational conditions, and how those conditions would be fulfilled to comply with Polish legislation.

### Results

Data collection for this study closed in the first quarter of 2015. Analyses examining the type of information reported by respondents are in progress, including comparisons of self-reported data with that available from electronic medical records and with the Danish National Prescription Registry. Analyses will be completed in 2015.

## Discussion

### Benefits and Challenges of Direct-to-Consumer Health Research Findings

Using direct reporting from consumers to study the safety of medicinal products allows researchers to address a myriad of research questions relating to everyday clinical practice, including treatment heterogeneity in population subgroups not traditionally included in clinical trials, like pregnant women, children, and the elderly. Internet-based studies such as this may also be useful for studying illicit drug use and other risky behaviors, since there is some evidence suggesting that patients will tell computers things that they might not tell health care professionals [7]. These studies can be supplemented with clinical validation and pharmacy prescription data, but direct-to-patient data collection may provide additional information about potentially harmful exposures that would not have been recorded elsewhere, and consequently would not be available to researchers. Nonetheless, there are a variety of administrative barriers, including obtaining informed consent for subjects participating by phone or Internet. The variations in informed consent requirements encountered here largely reflect challenges of recruitment without intervention of health care professionals, and are one of many complexities faced by Ethics Committees from use of emerging technologies [8].

### Added Complexities of Public-Private Partnerships

There were substantial barriers due to the nature of the funding structure, in addition to the challenges typically encountered in conducting direct-to-patient medical research. Public-private partnerships like this IMI project are becoming more prevalent as desirable funding mechanisms for research on the safety of medications and medical devices used in everyday clinical practice, for example, IMI Get Real [9] in Europe and the Food and Drug Administration’s efforts to build a postmarket National Medical Device Safety System in the United States [10]. In fact, at this time, the IMI is Europe's largest public-private initiative aiming to speed up the development of better and safer medicines for patients. With these large efforts come tremendous opportunities, but also substantial additional work relating to partnership governance, including shared liability. In this study, for example, assuring compliance with data protection requirements took about two years, which delayed data collection, reduced the overall time available for study conduct, and required substantial investment of legal and administrative time over and above any traditional research project. Moreover, most countries did not initially recognize the status of joint controller, arguing that only two partners had control of personal data, those who handled data collection and those who conducted study analyses. The concept that all parties to a research study must bear the full legal burden of being joint controllers, which includes accepting responsibility for legal damages regardless of culpability, needs updating. Fortunately, in this case all partners agreed to accept joint controller status, but refusal by one or more partners, or refusal by a country to accept that a person, agency, or institution had this status and/or to refuse a notification, could jeopardize other such collaborations.

The text of the proposed data protection regulation, which was endorsed by the European Parliament at its first reading in March 2014, if adopted into law, will do little to improve the situation [11]. The joint controller status still exists and although a single “competent” supervisory authority of the EU territory of the researcher’s main establishment can be requested to certify that the processing of personal data complies with the regulation, amendments to the proposed regulation require cooperation of supervisory authorities from other Member States. At the same time, supervisory authorities in disagreement with decisions are allowed the right of appeal to the European Data Protection Board. Uncertainty remains as to how this “cooperation” mechanism will operate to give much needed consistency. Moreover, the proposed regulation allows for multiple codes of conduct to be developed and approved by the supervisory authority of individual Member States and/or the European Commission, once again opening the door for disharmonized interpretations, now with much higher stakes since fines relating to failure to comply with the regulation can be as high as €100 million or 5% of annual worldwide turnover [11].

Data protection legislation is intended to allow freedom of movement of data, while protecting people from the theoretical harm of disclosure of personal data. This theoretical harm of disclosure of data that could be linked to an individual needs to be balanced against the potential for actual harm that could result from failure to identify safety signals in a timely fashion. Further, issues of data protection which require joint controller status to be shared among multiple parties may discourage participation, and might even drive health research away from regions of most interest to areas with potentially weaker protection of patient privacy and medication use that is quite different [4,12]. The potential financial consequences are considerable for an enterprise and may mean that companies or institutions may be reluctant to join consortia where the negligent actions of one partner could have such huge repercussions on the others, thus weakening the value of the public-private partnership investment.

## Abbreviations

EC: European Commission
EDPS: European Data Protection Supervisor
EU: European Union
IMI: Innovative Medicines Initiative
PROTECT: Pharmacoepidemiological Research on Outcomes of Therapeutics by a European ConsorTium
UK: United Kingdom
